# SSII-Evap: Simplified scheme to incorporate improved evapotranspiration estimates into the streamflow elasticity framework

**DOI:** 10.1016/j.mex.2019.03.021

**Published:** 2019-03-28

**Authors:** Urszula Somorowska

**Affiliations:** University of Warsaw, Faculty of Geography and Regional Studies, Department of Hydrology, Poland

**Keywords:** SSII-Evap: Simplified scheme to incorporate improved evapotranspiration estimates into the streamflow elasticity framework, Budyko-based evapotranspiration, Satellite-derived evapotranspiration, Bias correction, Runoff changes, Refined climate contribution, Human contribution

## Abstract

The streamflow elasticity concept based on the Budyko framework is widely used in hydrological impact assessment studies. However, in landscapes transformed by human activities, identification of climate contributions to runoff change is difficult due to changing surface properties of river basins. Here, a method is proposed to quantify the effects of changing vegetation cover and included them in the calculus. The simplified scheme to incorporate improved evapotranspiration estimates into the streamflow elasticity framework is introduced and named as “SSII-Evap” method. SSII-Evap allows for calculating runoff changes induced by climate taking into account: 1) changes in two climatic variables (precipitation and potential evapotranspiration) and 2) changes in land surface conditions responsible for varying actual evapotranspiration. The six-step procedure provides a focused guide for enhancing the original method.

• The SSII-Evap method introduces a bias correction to the original bivariate framework of streamflow elasticity to climate change.
• In contrast to the original method, SSII-Evap accounts for the influence of vegetation changes on actual evapotranspiration that is estimated from satellite-derived data.
• The elaborated customization is helpful for discriminating between climatic and human induced changes in mean annual runoff and is applicable to heavily modified river basins.

## Graphical abstract

**Figure:**
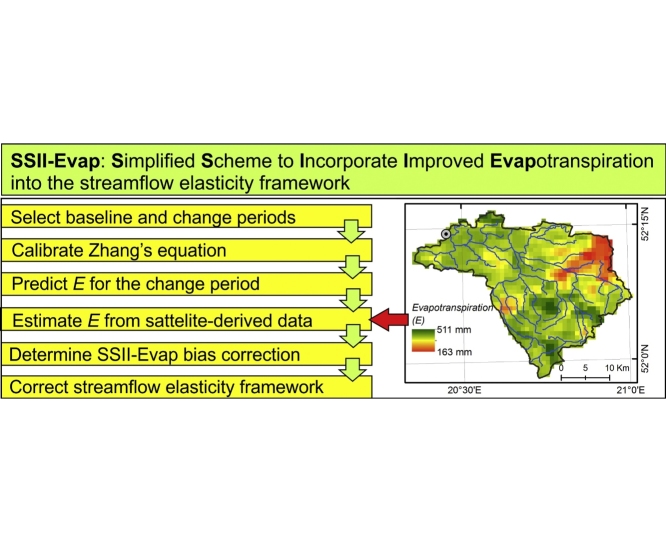

**Specifications Table**

**Table:** 

**Subject Area:**	Environmental Science
**More specific subject area:**	Climate and human-induced changes to river runoff
**Method name:**	SSII-Evap: Simplified scheme to incorporate improved evapotranspiration estimates into the streamflow elasticity framework
**Name and reference of original method:**	Climate elasticity of water resources (J. C. Schaake Jr., C. Liu, Development and application of simple water balance models to understand the relationship between climate and water resources. New Directions for Surface Water Modeling, Wallingford, IAHS Publ. 181: (1989) 343–352, http://hydrologie.org/redbooks/a181/iahs_181_0343.pdf)
	Sensitivity of runoff to long-term changes in precipitation without a change in vegetation (J.C.I. Dooge, M. Bruen, B. Parmentier, A simple model for estimating the sensitivity of runoff to long-term changes in precipitation without a change in vegetation. Adv. Water Resour. 23(2) (1999) 153–163, https://doi.org/10.1016/S0309-1708(99)00019-6)
**Resource availability:**	NA

## Method details

### Method objectives

**Fig. 1:**
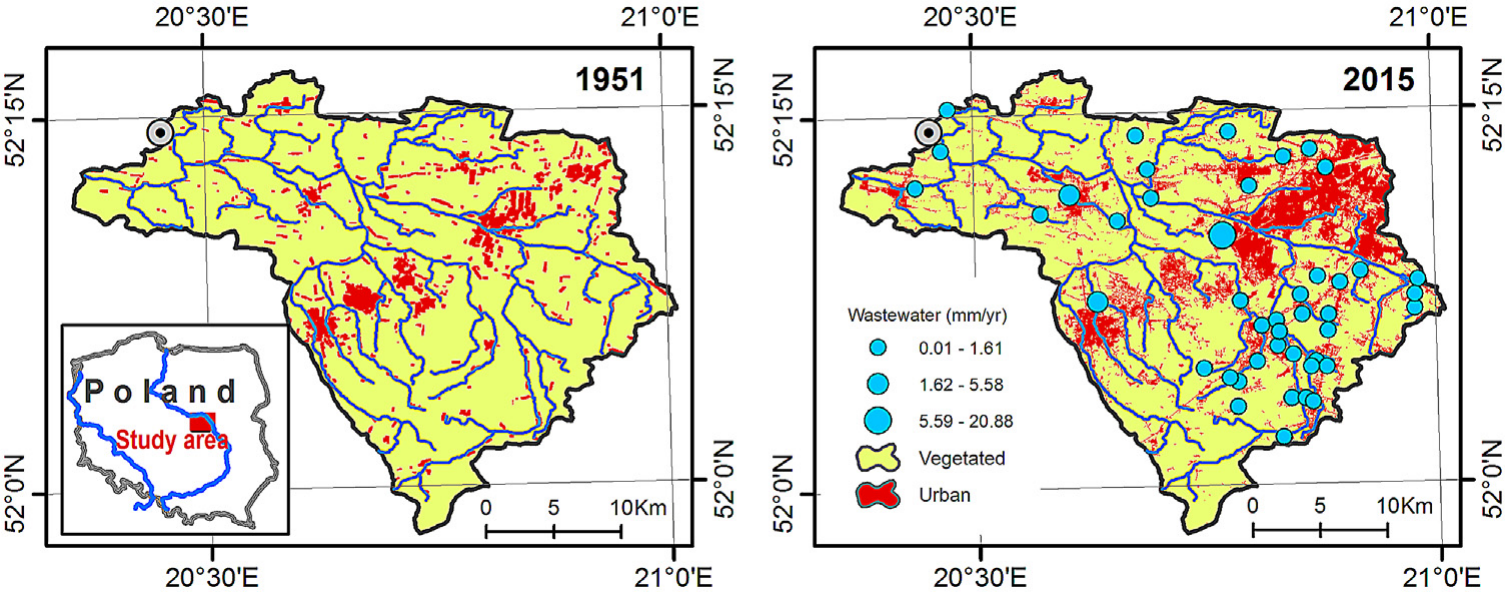
Changes in land cover in the Utrata River Basin, between 1951 and 2015. Rapid urban growth significantly decreased the vegetated area, hence diminished the actual evapotranspiration. An increase in inflow from the wastewater treatment plants is a result of increased municipal and industrial water use imported to the hydrological system from external resources.

A simplified scheme to incorporate improved evapotranspiration estimates into the streamflow elasticity framework is proposed and named as “SSII-Evap” method. The main objective is to include the effect of long-term changes in vegetation on mean annual runoff, and to isolate them from other temporally variable properties of a river basin. The elaborated scheme improves runoff attribution methods that are based on the water balance and streamflow elasticity concepts, which are nowadays commonly applied approaches in hydrology. The SSII-Evap method helps to discriminate between climatic and human-induced changes to mean annual runoff. Based on this method, satellite-derived evapotranspiration estimates are assimilated into the calculus for adjusting the contribution of a varying climate to runoff change. The customization is designed to work as a bias correction of the bivariate streamflow sensitivity framework introduced by Dooge et al. in 1999 [1]. The adapted framework is applicable to heavily modified river basins under the influence of different factors that act simultaneously. This manuscript is meant to provide a step-by-step explanation of how to apply the elaborated adaptation used previously in Somorowska and Łaszewski [2] where it was described with fewer details. The main steps of the SSII-Evap method are presented using an example of the 727 km^2^ mesoscale Utrata River Basin (URB) in central Poland, which is influenced by a varying humid temperate climate, wastewater inflow, and urbanization (Fig. 1).

### Existing streamflow elasticity method based on Budyko framework

The elasticity concept was introduced in hydrology by Schaake and Liu [3] to quantify the elasticity of water resources to climatic change. Dooge et al. [1] further contributed to the concept by proposing the bivariate framework of streamflow sensitivity to climate change. It was assumed that streamflow changes due to climate variability is the sum of changes caused by precipitation (P) and potential evapotranspiration ($E_{P}$):

(1) $$\Delta Q_{clim}=\Delta Q_{P}+\Delta Q_{E_{P}}$$

In this approach, runoff changes are computed assuming stability of the watershed vegetation and soil system over long-term periods. Using the coefficients of streamflow elasticity, Eq. (1) is rewritten as:

(2) $$\Delta Q_{clim}=e_{Q,P}\Delta P+e_{Q,Ep}\Delta E_{P}$$

where $e_{Q,P}$ is the absolute precipitation elasticity coefficient, and $e_{Q,Ep}$ is the absolute potential evapotranspiration elasticity coefficient [4]. The absolute runoff elasticity to precipitation is defined as the ratio between mm change of runoff (Q) by mm change of precipitation (P). Consequently, absolute runoff elasticity to potential evapotranspiration is defined as the ratio between mm change of runoff (Q) by mm change of potential evapotranspiration ($E_{P}$). Hence, the elasticity coefficients measure the responsiveness of runoff to changes in the specific climate variable. They can be derived analytically from the Budyko-type equation by calculating the first derivatives of runoff. Using the Budyko hypothesis, a couple of different mathematical formulas of mean annual water balance model were developed, and among them is Zhang’s equation [5], which is used in this study:

(3) $$\frac{E}{P}=\left[1+w\left(\frac{E_{P}}{P}\right)\right]/\left[1+w\left(\frac{E_{P}}{P}\right)+\frac{P}{E_{P}}\right]$$

where E, P and $E_{P}$ are mean annual values of actual evapotranspiration, precipitation, and potential evapotranspiration, respectively. In this theoretical framework, the evaporative ratio (E/P) is related to the aridity index ($E_{P}/P$) through the parameter *w* that reflects river basin properties. Eq. (3) is rewritten as:

(4) $$Q(P,E_{P},w)=P-P\left[1+\left(\frac{E_{P}}{P}\right)\right]/\;\left[1+w\left(\frac{E_{P}}{P}\right)+\frac{P}{E_{P}}\right]$$

and then the sensitivity of runoff to varying precipitation and potential evapotranspiration is derived as proposed by Li et al. [6]:

(5) $$e_{Q,P}=(1+2\phi +3w\phi^{2}\;)/{\left(1+\phi +w\phi^{2}\right)}^{2}$$

(6) $$e_{Q,E_{P}}=-\left(1+2w\phi \right)/{\left(1+\phi +w\phi^{2}\right)}^{2}$$

where ϕ is the aridity index and is equal to $E_{P}/P$. The parameter w and the streamflow elasticity coefficients are estimated using mean annual hydroclimatic data for the baseline period. In such a case, the climate contribution to runoff changes in the change period is calculated as a forward approximation with an assumption that specific features of the river basin (physical characteristics and vegetation-soil properties) remain unchanged (Δw=0). Whilst it is safe to consider the geologic and topographic properties as nearly constant over long-term periods, structural and compositional changes in vegetation cover are evident over much shorter time scales. As evapotranspiration fluxes are highly sensitive to vegetation cover changes, a refinement to the climate contribution calculus to runoff changes is proposed.

### Correcting the streamflow elasticity framework for vegetation changes

The SSII-Evap method was developed as a refinement of the Dooge approach [1]. The re-defined climate contribution to streamflow change is expressed by a three-component equation:

(7) $$\Delta Q_{clim\_corrected}=e_{Q,P}\Delta P+e_{Q,Ep}\Delta E_{P}+\Delta Q_{clim,\;E}$$

The third component of Eq. (7), $\Delta Q_{clim,\;E}$, provides a bias correction of $\Delta Q_{clim}$ and was designed to correct the actual evapotranspiration predicted using a Budyko-type equation. In the first step, a single-parameter mean annual water balance model (Eq. (3)) is calibrated for the baseline period, and is then used to calculate actual evapotranspiration in the change period as if vegetation properties remained constant. Then, the predicted value of the Budyko-based evapotranspiration ($E^{model}$) is checked against the satellite-derived estimate ($E^{sat}$). The SSII-Evap bias-correction is defined as:

(8) $$\Delta Q_{clim,E}=E^{model}-E^{sat}$$

Recently, development of numerous remote sensing-based evapotranspiration algorithms provides a range of evapotranspiration products. One of the few available and operational products is the Simplified Surface Energy Balance (SSEBop) evapotranspiration [7] that is applied in this study. Globally available monthly estimates of actual evapotranspiration are accessible from the United States Geological Survey (USGS) Data Portal (https://earlywarning.usgs.gov/fews/product/460). Multi-year annual actual evapotranspiration for a change period can be estimated from the spatiotemporal patterns in evapotranspiration data that are included in Eq. (8). The continuous development of remote sensing techniques makes it possible to use ever newer data. With the SSII-Evap method, SSEBop data is interchangeable with any other future satellite-based product that accurately captures evapotranspiration variability.

The SSII-Evap bias correction is close to zero under constant climate conditions during which vegetation cover is relatively stable. In such a case, the relation between the evaporative ratio and the aridity index for the change period is close to that for the baseline period. If changes in vegetation cover occur, the bias-correction can take on either a positive or negative value. When $E^{sat}$ is higher than $E^{model}$, the value of the bias-correction is negative. Simultaneously, negative bias correction denotes a reduction of climate contribution to the runoff change. Contrastingly, positive bias correction indicates an increase in mean annual runoff, and consequently, an increase in the climate contribution to the runoff change.

The SSII-Evap method improves the actual evapotranspiration for the change period. Simultaneously, it can contribute to discrimination between changes in runoff induced by changes in vegetation and those induced by other changes in land cover, for example, those connected to sprawling urbanization. By using this approach, changes caused by vegetation are incorporated into the climate contribution, whereas the other land cover changes account for human contribution, specifically those induced by land management practices.

### Including a corrected climate contribution into the total runoff change

The SSII-Evap corrected climate contribution is incorporated into the total runoff change equation as:

(9) $$\Delta Q_{tot}=\Delta Q_{clim\_corrected}+\Delta Q_{hum}$$

where $\Delta Q_{clim\_corrected}$ is the change in river runoff caused by climate variability as defined by Eq. (7), and $\Delta Q_{hum}$ is river runoff change caused by changing properties of a river basin due to human activities. Simultaneously, total runoff variation, $\Delta Q_{tot}$, is calculated as:

(10) $$\Delta Q_{tot}=Q_{obs,CP}-Q_{obs,BP}$$

where $Q_{obs,CP}$ and $Q_{obs,BP}$ represent the mean annual runoff for the change and baseline periods, respectively. Hence, the human contribution to the runoff change is estimated as:

(11) $$\Delta Q_{hum}=Q_{obs,CP}-Q_{obs,BP}-\Delta Q_{clim\_corrected}$$

In the case of human-influenced changes caused by wastewater inflow and sprawling urbanization, the human contribution to runoff changes is expressed as:

(12) $$\Delta Q_{hum}=\Delta Q_{WWTP}+\Delta Q_{urb}$$

where $\Delta Q_{WWTP}$ is the change caused by wastewater treatment plant (WWTP) inflow and $\Delta Q_{urb}$ is the change caused by urbanization. Where the mean annual wastewater inflow is known, the mean annual change in runoff due to urbanization is derived as a residual:

(13) $$\Delta Q_{urb}=\Delta Q_{tot}-\Delta Q_{clim\_corrected}-\Delta Q_{WWTP}$$

where $\Delta Q_{urb}$ is attributed to urban land fragmentation and associated land management practices. Finally, the relative contribution of each of the primary factors recognized is calculated as:

(14) $$\eta_{clim}=\frac{\left|\Delta Q_{clim\_corrected}\right|}{\left|\Delta Q_{clim\_corrected}\right|+\left|\Delta Q_{WWTP}\right|+\left|\Delta Q_{urb}\right|}\cdot 100\text{\%}$$

(15) $$\eta_{hum}=\frac{\left|\Delta Q_{WWTP}\right|+\left|\Delta Q_{urb}\right|}{\left|\Delta Q_{clim\_corrected}\right|+\left|\Delta Q_{WWTP}\right|+\left|\Delta Q_{urb}\right|}\cdot 100\text{\%}$$

The six-step procedure to determine and apply the SSII-Evap bias correction for a specific river basin is detailed in the following section.

## Step 1: Select baseline and change periods

**Fig. 2:**
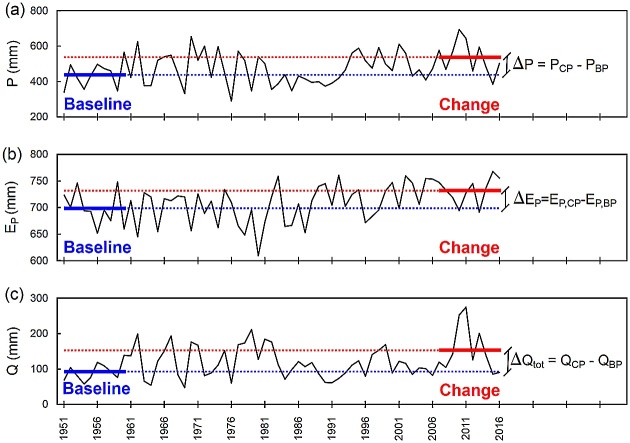
Estimating mean annual values of hydrometeorological variables for the change period (CP) and baseline period (BP).

First, the multi-year baseline and change periods are selected. The baseline period serves as the reference period from which runoff deviation in the change period is calculated. Ideally, the baseline should cover a multiyear period with negligible human impact so that the river basin’s hydrological functioning is identified under near natural conditions. Such a choice ensures that runoff formation is due to varying climate and is not a result of unstable river basin properties. Vegetation and land cover in this period should not have undergone significant changes. In contrast, the primary human impacts should be recognized in a change period. In case of the URB, wastewater inflow and urbanization were the main factors contributing to runoff changes. Wastewater inflow is an identifiable quantity. In this example, the baseline period is based on a 10-year (1951–1960) period of stability and the change period covers the latest 10-year period available (2007–2016). Based on the mean annual values of precipitation, potential evapotranspiration, and observed runoff for the baseline and change periods (Table 1), changes in hydrological variables, namely ΔP, $\Delta E_{P}$, and $\Delta Q_{tot}$ were calculated (Fig. 2). ΔP and $\Delta E_{P}$ are required as input to Eq. (2), whereas $\Delta Q_{tot}$ is needed to solve Eq. (11).

**Table 1:** Mean annual values of climatic and hydrologic variables for baseline and change periods.

Climatic/hydrologic variable	Baseline period	Change period	Change in climatic/hydrologic variable
(mm)	(1951–1960)	(2007–2016)	(mm)
Precipitation	438.45	537.41	98.96
Potential evapotranspiration	698.90	731.88	32.98
Actual evapotranspiration	346.25^a^	379.45^b^ 400.99^c^	33.20
Observed runoff	92.2	153.3	61.1
Wastewater inflow	0.0	36.4	36.4
Naturalized runoff	92.2	116.9^d^ 136.4^e^	24.7

a Estimated as the difference between precipitation and naturalized runoff.

b Estimated from the SSEBop data.

c Estimated by using Eq. (16).

d Estimated as the difference between observed runoff and wastewater inflow.

e Estimated by using Eq. (4).

## Step 2: Calibrate Zhang’s equation

The parameter *w* of Zhang’s model is calibrated for the baseline period assuming that storage variation in the river basin over a multiyear period can be disregarded. The mean annual values of P and $E_{P}$ for the baseline period are indicated in Fig. 2 by a blue solid line. In Eq. (3), mean annual E is substituted for the difference between the mean annual values of precipitation and runoff, and then the parameter *w* is fit. Further, the runoff elasticity coefficients are calculated from Eqs. (5) and (6) as required to calculate $\Delta Q_{clim}$.

## Step 3: Predict evapotranspiration for the change period

**Fig. 3:**
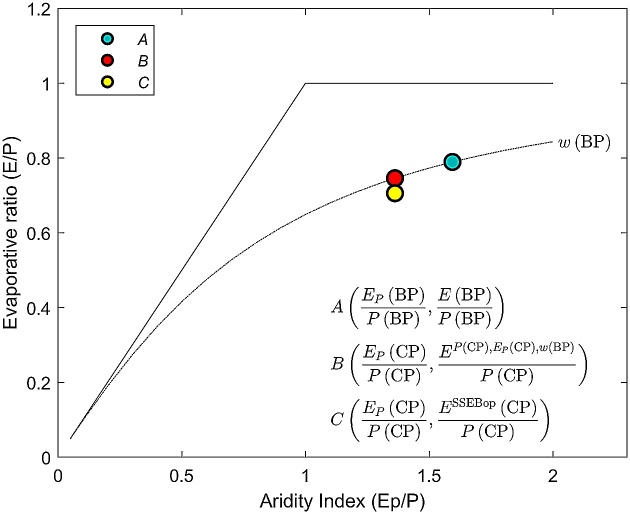
Predicting evaporative ratio for the change period. Point *A* represents baseline period conditions, point *B* stands for change period conditions without SSII-Evap bias correction, and point *C* depicts change period conditions with SSII-Evap bias correction.

The mean actual evapotranspiration during the change period is calculated as:

(16) $$E^{P(CP),E_{p}(CP),w(BP)}=P\left[1+w\left(\frac{E_{P}}{P}\right)\right]/\left[1+w\left(\frac{E_{P}}{P}\right)+\frac{P}{E_{P}}\right]$$

where P(CP) and $E_{p}(CP)$ are the mean annual values of precipitation and potential evapotranspiration for the change period, respectively, and w(BP) is the *w* value calibrated for the baseline period. Mean annual values of climatic variables for the change period are indicated in Fig. 2 by a solid red line. The value of $E^{P(CP),E_{p}(CP),w(BP)}$, described in Eq. (8) as $E^{model}$, is a proxy for actual evapotranspiration in the change period assuming that vegetation controls the hydrological system as during baseline period (Fig. 3). During the baseline period the relationship between the evaporation ratio (E/P) and the climatic dryness index ($E_{P}/P$) is represented by point *A*, which is located on the Budyko-type curve of (BP). For the change period, the relationship between the evaporation ratio and the climatic dryness index is predicted to shift to point *B* assuming that it is still located on a curve representing the baseline conditions. In reality, the point representing the change conditions is deviated from point *B* since changes in actual evapotranspiration are caused not only by climatic variables, but also by any changes in vegetation. Therefore, in the next steps, $E^{P(CP),E_{p}(CP),w(BP)}$ is checked against satellite-derived evapotranspiration and the SSII-Evap correction is determined.

## Step 4: Estimate evapotranspiration from satellite-derived data

**Fig. 4:**
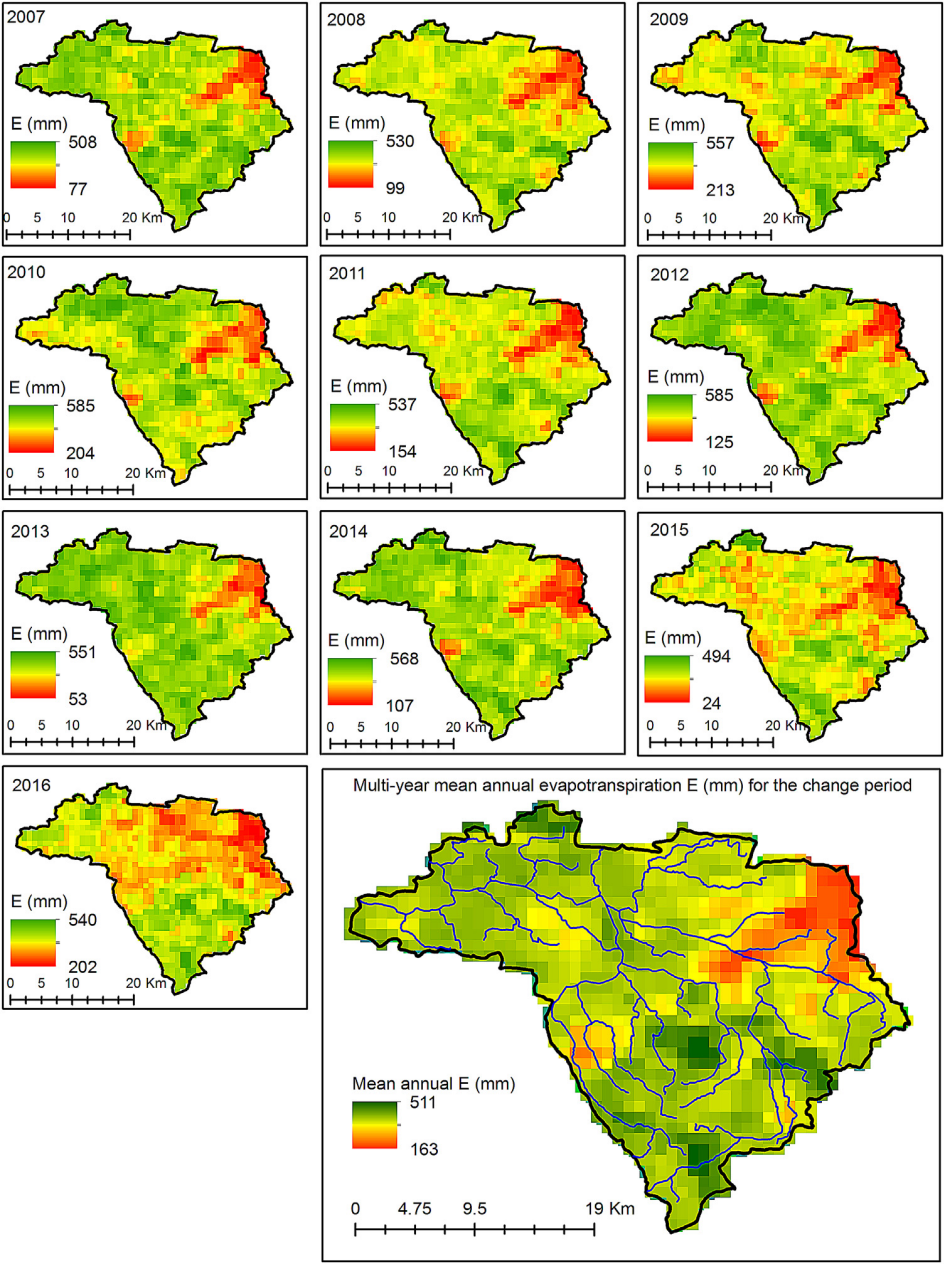
Deriving mean annual evapotranspiration from the SSEBop data. From monthly satellite-based data, annual raster images are prepared and then raster image of multi-year mean annual evapotranspiration for change period is derived. Based on that, areal-weighted multi-year mean evapotranspiration is calculated as a single value for the river basin.

Monthly raster images of actual evapotranspiration are acquired for the change period from the United States Geological Survey (USGS) Data Portal (https://earlywarning.usgs.gov/fews/product/460). All images are cropped to the river basin boundary using ArcGIS 10.5 software [8]. For each water year, twelve monthly images are overlaid and summed to a separate raster image representing spatial patterns of annual evapotranspiration (Fig. 4). Then, a multiyear mean annual raster image is derived, and finally, area-weighted mean annual evapotranspiration, $E^{SSEBop(CP)}$, is calculated. The SSEBop data provide reliable evapotranspiration estimates over wide-areas and work well for both cropland [9] and urban areas [10].

## Step 5: Determine SSII-Evap bias correction

The SSII-Evap bias correction is calculated according to Eq. (8) as:

(17) $$\Delta Q_{clim,E}=E^{P(CP),E_{p}(CP),w(BP)}-E^{SSEBop(CP)}$$

The first element of Eq. (17), $E^{P(CP),E_{p}(CP),w(BP)}$, is calculated in step 3, and the second element, $E^{SSEBop(CP)}$, is estimated from the satellite-based data, described in step 4.

## Step 6: Include SSII-Evap bias correction into calculus of the total runoff change

The steps 1–5 described how to identify the two components of Eq. (9), namely $\Delta Q_{tot}$ and $\Delta Q_{clim\_corrected}$. Next, it is possible to extract runoff changes due to urbanization as a residual:

(18) $$\Delta Q_{urb}={\Delta Q}_{tot}-{(e}_{Q,P}\Delta P+e_{Q,Ep}\Delta E_{P})-\Delta Q_{clim,E}-\Delta Q_{WWTP}$$

where $\Delta Q_{WWTP}$ is estimated based on data acquired from wastewater treatment plants. Finally, the relative contributions of each component to the total change in runoff are calculated separately as a percentage of the total change in runoff or by grouping the climate and human factors as shown in Eqs. (14) and (15).

### Validation of the SSII-Evap method

Using the example of the URB (Fig. 1), mean annual values of hydroclimatic variables were derived and presented in Table 1. The calibrated value of parameter *w*, representing the river basin’s characteristics in Zhang’s equation, was estimated for the baseline period and was equal to 0.8506. The absolute elasticities for precipitation and potential evapotranspiration amounted to 0.5449 and -0.1630, respectively. The value of the SSII-Evap bias correction, $\Delta Q_{clim,\;E}$, was equal to 21.54 mm, constituting approximately 6% of the $E^{SSEBop(CP)}$. Predicted actual evapotranspiration, $E^{model}$, was greater than $E^{SSEBop(CP)}$. Greater $E^{model}$ is explained by the changes in vegetation and land cover that took place over the decades between the baseline and change periods in the URB [2]. Urban growth occurs at the expense of the forested and agricultural land areas, which are much more evaporatively active than partially impervious urban and suburban surfaces. Evapotranspiration of the sprawling urban system was greater in an absolute sense than during the baseline period, but was relatively smaller than that predicted for the system with unchanged surface conditions (Table 1).

The elasticity based approach customized with the SSII-Evap method was validated using the water balance approach. First, mean annual naturalized runoff was predicted for the change period as:

(19) $$Q_{nat,CP}=Q^{P(CP),E_{p}(CP),w(BP)}+\Delta Q_{clim,E}$$

where $Q^{P(CP),E_{p}(CP),w(BP)}$ was calculated according to Eq. (4). Then, the contribution of climate to the total change in runoff was calculated as:

(20) $$\Delta Q_{clim\_corrected}=Q_{nat,CP}-Q_{obs,BP}$$

Following this, the human contribution was estimated as:

(21) $${\Delta Q}_{hum}=Q_{obs,CP}-Q_{nat,CP}$$

Finally, the relative contribution of each factor was calculated as a percentage of the total change in runoff according to Eqs. (14) and (15). Validation results using the water balance approach are presented in Table 2. Compared to the runoff contributions calculated with the corrected streamflow elasticity framework, the water balance-based approach produced a difference in a climate and human contributions of ± 0.2%.

**Table 2:** Validation of the corrected streamflow elasticity framework by a water balance approach.

Contribution	Elasticity		Water balance		Difference
	$\text{Δ}Q_{X,el}$ (mm)	$\eta_{X,el}$ (%)	$\text{Δ}Q_{X,WB}$ (mm)	$\eta_{X,WB}$ (%)	$\eta_{X,el}-\eta_{X,WB}$ (%)
$\text{Δ}Q_{clim\_corrected}$	70.09	46.1	65.76	45.9	0.2
$\text{Δ}Q_{hum}$:	−8.99	53.9	−4.66	54.1	−0.2
$\text{Δ}Q_{WWTP}$	36.4	24.0	36.4	25.4	
$\text{Δ}Q_{urb}$	−45.39	29.9	−41.06	28.7	

In order to understand the redefined framework more completely, the calculated contributions of climate and human impacts with the SSII-Evap method were compared with results estimated with the original bivariate elasticity framework (Table 3). The customized method indicated that the contribution of climate was slightly greater than with the original method. The positive SSII-Evap bias correction value was caused by lower evapotranspiration in the urbanized environment than when it was naturally vegetated land. Relatively lower evapotranspiration contributes to increased runoff. However in this case, other changes (those associated with land management practices, i.e., discharge from the Okecie airport drainage system to the adjacent river basin) contributed to significant runoff reduction (Table 2). Consequently, the human contribution estimated with the SSII-Evap method appeared slightly lower than when estimated with the original elasticity approach. While the contributions evaluated by the customized and original methods do not show substantial percentage differences, it was demonstrated that long-term changes in vegetation have resulted in runoff increases of 21.54 mm annually, which is equivalent to approximately 15 million cubic meters per year. This highlights the importance of identifying and quantifying evapotranspiration for improved management in urban system design and water allocation. Remote sensing technologies provide powerful sources of information that are quickly and efficiently incorporated into the water management planning and sustainable landscape management.

**Table 3:** Contribution of climate variability and human disturbance to the runoff change according to modified and original elasticity frameworks.

Contribution	Elasticity with SSII-Evap		Elasticity (original)		Difference
	$\text{Δ}Q_{X,el\_SSII}$ (mm)	$\eta_{X,el\_SSII}$ (%)	$\text{Δ}Q_{X,el}$ (mm)	$\eta_{X,el}$ (%)	$\eta_{X,el\_SSII}-\eta_{X,el}$ (%)
Climate	70.09	**46.1**	48.55	**44.6**	1.5
Human:	−8.99	**53.9**	12.55	**55.4**	−1.5
WWTP	36.40	24.0	36.40	33.5	
urbanization	−45.39	29.9	−23.85	21.9	

## Funding

This research was supported by the Faculty of Geography and Regional Studies at the University of Warsaw in Poland (grant no. 501-D119-64-0180200-01-802).

## Conflicts of interest

The author declares no conflicts of interest.
